# Knowledge, use and management of native wild edible plants from a seasonal dry forest (NE, Brazil)

**DOI:** 10.1186/1746-4269-9-79

**Published:** 2013-11-26

**Authors:** Margarita Paloma Cruz, Nivaldo Peroni, Ulysses Paulino Albuquerque

**Affiliations:** 1 Biology Department, Laboratory of Applied and Theoretical Ethnobiology, Botany Area, Federal Rural University of Pernambuco, Pernambuco, Brazil; 2 Colombian Society of Ethnobiology, Bogotá, Colombia; 3 Ecology and Zoology Department (ECZ), Federal University of Santa Catarina, Santa Catarina, Brazil

**Keywords:** Ethnobotany, Human ecology, Consumption, Food plants, Rural communities

## Abstract

**Background:**

Despite being an ancient practice that satisfies basic human needs, the use of wild edible plants tends to be forgotten along with associated knowledge in rural communities. The objective of this work is to analyze existing relationships between knowledge, use, and management of native wild edible plants and socioeconomic factors such as age, gender, family income, individual income, past occupation and current occupation.

**Methods:**

The field work took place between 2009 and 2010 in the community of Carão, Altinho municipality, in the state of Pernambuco in northeastern Brazil. We conducted semi-structured interviews with 39 members of the community regarding knowledge, use and management of 14 native wild edible plants from the Caatinga region, corresponding to 12 vegetable species. In parallel, we documented the socioeconomic aspects of the interviewed population (age, gender, family income, individual income, past occupation and current occupation).

**Results:**

Knowledge about edible plants was related to age but not to current occupation or use. Current use was not associated with age, gender or occupation. The association between age and past use may indicate abandonment of these resources.

**Conclusion:**

Because conservation of the species is not endangered by their use but by deforestation of the ecosystems in which these plants grow, we suggest that the promotion and consumption of the plants by community members is convenient and thereby stimulates the appropriation and consequent protection of the ecosystem. To promote consumption of these plants, it is important to begin by teaching people about plant species that can be used for their alimentation, disproving existing myths about plant use, and encouraging diversification of use by motivating the invention of new preparation methods. An example of how this can be achieved is through events like the “Preserves Festival”.

## Background

The use of wild edible plants is an ancient tradition that has been increasingly neglected, especially in industrialized societies and those communities in which the proximity of industrialized populations increasingly threatens the perpetuation of this knowledge despite its antiquity and the fact that it meets one of the basic human needs [1].

One possible reason to abandon this tradition is that the use of wild plant species is considered to be synonymous with poverty [2-5], with the consumption of more industrialized food being considered more prestigious, at least in Latin American countries, such as Argentina [2,3,6], Brasil [5], and Colombia (direct observation), as well as some African countries, such as Lesoto [7] and Sudan [4,8].

A large number of studies on wild edible plants exist [1-9] many of which attempt to understand the patterns in the distribution of the knowledge associated with the use of these plants according to the age and gender of the consumers. Arias-Toledo et al. [3] compared the knowledge and use related to medicinal and food plants in the argentinian Chaco, finding no significant difference in the number of edible plants known by people of different age or gender. An opposite result was found by Ladio y Lozada [9], who examined differences in use and knowledge among members of Mapuche communities, according to the environments in which these plants are collected in the Patagonia region of Argentina. The authors identified four well-differentiated collection locations where different usage patterns and knowledge of wild edible plants were observed, with the most conserved sites being those exhibiting less differentiation with respect to the age and gender parameters. Thus, all plants known to be edible are actually consumed in these environments.

Reyes-Garcia et al. [1] worked with two human communities in the bolivian Amazon and found that the use and knowledge of wild edible plants is better conserved in the community that is located farther away from an urban center. In this community, both variables (knowledge and use) are statistically correlated, which does not occur in the community located closer to the urban center.

In Brazil some studies on edible plants have been conducted [10-13]. A noteworthy study was carried out by Nascimento et al. [5,10], who investigated the known and consumed edible species, both native and exotic, in two rural communities in northeastern Brazil, finding that, although the native species were not numerous, they were the most commonly cited. In addition, the authors did not found significant differences in the knowledge based on age.

Several researchers have studied human actions that intervene in the evolution of useful plants [14-18] and have proposed four types of *in situ* management practices that humans can impose on plant populations, for supporting survival of individual plant species with desirable traits. These practices include systematic collection, tolerance, protection and promotion of individuals plant species.

Collecting edible plants could be considered an *in situ* form of management, as long as the collected elements belong to populations presenting desirable phenotypes such that this selective leads to the differentiation of regions or groups of individual plants that are preferred for the collection of these plants [14,16,18]. Tolerance can be considered the non-removal of plants that may offer some type of useful product for people within areas devoted to planting or cultivating other natural resources [11,14,17].

Protection implies deliberate elimination of competitors and predators of useful plants, as well as trimming, protecting against unfavorable climatic conditions, and/or nutrient or mineral supplement addition for better plant development [14,16,17]. Promotion considers strategies aimed at increasing the population density of desired species through intentional planting of seeds or vegetative structures of a species in wild areas [17].

Agre et al. [19] conducted a study in Uganda that evaluated the influences of gender, degree of education, amount of land dedicated to agriculture and occupation on attitudes of community members toward planting native fruit species. They found significant differences in attitudes based on gender; women tended to desire more native fruit plants than men. Ekué et al. [18] found that management practices to increase the number of ackee (*Blighia sapida*) plants also varied by gender, with women more commonly in charge of this type of activity.

The objective of this study was to examine the patterns related to the knowledge, use, and management of native wild edible plants in a Caatinga area, proposing the following hypothesis: socioeconomic factors (age, gender, family income, individual income, past occupation and current occupation) are related with knowledge, use and management of native wild edible plants by people.

To investigate our hypothesis, we evaluated the relationships between socioeconomic factors and knowledge indicators as well as the use and management of native wild edible plant species presented in Table 1[9]. According to the findings of other studies, we anticipated that the knowledge and usage of native wild edible plants were related, as reported by Ladio y Lozada, Arias-Toledo [3] and Nascimento et al. [5]. We also expected no relationship between age and knowledge of native wild edible plants, nor between monthly income (family and personal) and knowledge of these plants. Additionally, we expected to find a relationship between occupation and knowledge and the use of the plants and none between gender and plant use, as found by Arias-Toledo et al. [3]. A relationship between management and gender was expected, as reported by other authors [18,19].

**Table 1:** Studied variables employed to analyze the relation between socioeconomic factors and knowledge, use and management of native wild edible plants from a local community in NE Brazil

**Socioeconomic factors**	**Selected indicators to analyse knowledge, use and management**	
Age	Knowledge	Number of known edible items
Gender	Use	Number of edible items currently used
Monthly family income		Number of edible items used in the past
Monthly individual income		Number of uses
Past occupation	Management	Number of forms of preparation
Current occupation		Do you go out exclusively for gathering these items?
		When you are preparing the field for planting and you find any of these plants, which of them would you tolerate?

In addition to the variables presented in Table 1, other questions were asked that were analyzed in a qualitative manner, to provide better understanding of plant knowledge, usage and management patterns. These questions were the following: Which of these plants were consumed only once? Why did you not continue consuming them? Do you consume these plants with the same frequency as before? Why or why not? What are some ways of preparing these plants? Where is the most common harvesting location? Which is the preferred harvesting location? Do you plant or grow some of these plants in any way? How? Would you change something in these plants?

## Methods

### Study area

This study was part of the project “Caatinga Plant Resources: Use, Diversity and Conservation”, which was approved by the Ethics Committee for Research Involving Human Subjects of the Health Sciences Center of Pernambuco Federal University (register number 238–06). The objectives of the project, along with the methodological procedures employed, were previously presented and discussed with the members of the community, who were then asked to agree to participate and to sign a Free and Transparent Consent form (TCLE, according to resolution 196/96 of the National Health Council) [20-23].

The study area is located in northeastern Brazil in the state of Pernambuco, Altinho municipality, in the community of Carão (8° 29′ S and 36° 03′ W). The community is located 16 km from the municipal center, and the municipality is located 160 km from the state capital [24]. The region is located in a Caatinga ecosystem, which is characterized by its hot and semiarid climate, its unimodal rain regime and by strong rainfall seasonality, with a marked semiarid period of seven to nine months and a rainy period associated with torrential rains [25]. These climatic conditions determine that the local vegetation is composed of deciduous and xerophyte species, including ‘permanent’ species, which manage to survive even under drought conditions, and ‘periodic’ species, which emerge only in the rainy season [25].

The investigated human community is typically rural and is composed of approximately 189 inhabitants, of which 112 are older than 18 years; 60% of the inhabitants are women and 40% are men, according to information provided by the local health post [20]. The main economic activities of the members of the community are the production of bovine and caprine cattle, along with subsistence agriculture consisting of corn and bean cultivation, the surplus products of which are sold at the weekly market in the urban area of the municipality [11,26].

Heads of household who could name at least four edible plant uses in general interviews conducted by another member of the Applied Ethnobiology Laboratory [11,20,22,23,26,27] were included in the survey employed in this study. Using this criterion, 44 people were selected, of which 39 were actually interviewed (27 women and 12 men); the other five either did not want to continue participating in the study or left the community.

From the general interviews, the 12 most commonly cited native wild edible plant species were selected for this study (see Table 2). Because both the fruits of *Pilosocereus pachycladus* subsp *pernambucoensis* (Ritter) Zappi (local common name: facheiro) and *Spondias tuberosa* Arruba (local common name: imbu) and other parts of these plants are consumed (cladode and tubercle, respectively), 14 edible items were considered in total.

**Table 2:** Native wild edible plants examined in this study addressing the knowledge, use, and management of native wild edible plants from a local community in NE Brazil

**Local common name**	**Family**	**Species**
Batinga	Myrtaceae	*Eugenia* sp.
Cana de macaco	Marantaceae	*Maranta gibba* Sm
Coco catolé	Arecaceae	*Syagrus cearensis* Noblick
Facheiro	Cactaceae	*Pilosocereus pachycladus* subsp. *pernambucoensis* (Ritter) Zappi
Imbu	Anacardiaceae	*Spondias tuberosa* Arruda
Incó	Brassicaceae	*Neocalyptrocalyx longifolium* (Mart.) X. Cornejo & H.H. Iltis
Jatobá	Fabaceae	*Hymenaea courbaril* L.
Mandacaru	Cactaceae	*Cereus jamacaru* DC.
Manofê	Apocynaceae	*Mandevilla tenuifolia* (J.C. Mikan) Woodson
Pirim	Myrtaceae	*Psidium schenckianum* Kiaersk
Trapiá	Brassicaceae	*Crateva tapia* L.
Ubaia	Myrtaceae	*Eugenia pyriformis* Cambess.

Plant material was collected during fieldwork and was stored in the UFP-Geraldo Mariz Herbarium of Pernambuco Federal University; some duplicate samples were sent to the HUEFS herbarium of the State University of Feira de Santana.

### Data collection and analysis

Before interviews were performed, informal conversations were conducted with 30 inhabitants of the community with the objective of determining which type of information needed to be collected. Based on these conversations, semi-structured interviews were designed and conducted between the months of September 2009 to May 2010. In these interviews, socioeconomic information was collected for the 39 people surveyed as well as information regarding their knowledge of the selected 14 edible items (we considered edible items to be not only the species but also the parts used), along with their current and past usage, form of preparation, collecting locations, type of management, variations in use over time and, if applicable, the reasons why these items have not been consumed.

To guarantee that the interviewer and the interviewees were referring to the same species and to assist people in remembering the edible items about which they were surveyed, illustrative photographs of the organs of the plants involved in each question were employed [28].

The percentage of responses obtained for each question was calculated. To evaluate whether relationships exist between the different socioeconomic factors (age, gender, monthly family income, monthly individual income, past occupation, current occupation) and knowledge, use and management variables investigated, we performed linear regressions for the normally distributed continuous variables and correlation analysis when there were no dependent and independent variables, and we used contingency tables for the discrete variables. All analyses were conducted using BioEstat 5.0 software [29].

**Figure 1:**
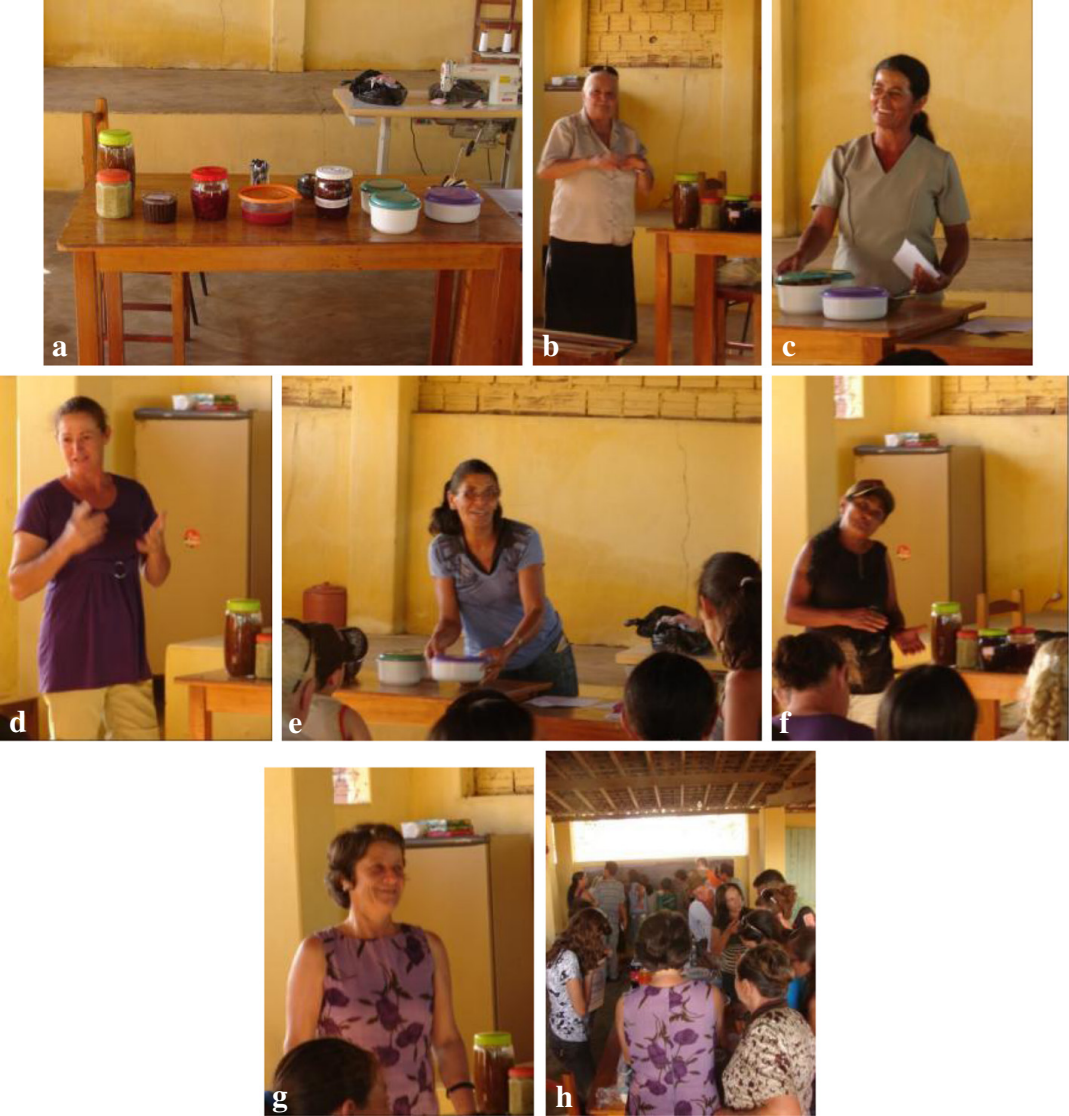
**Photographs of the “Preserves Festival” held at the end of the study with the inhabitants from a local community in NE Brazil. a**. Participating preserves. **b-g**. Sweet jam makers presenting their products and explaining how to prepare them. **h**. Members of the community tasting the preserves.

After the study ended, the participants were summoned to a community event (the “Preserves Festival”), with the intention to 1) inform them about the results obtained; 2) hear their reflections on these results; and 3) promote use of wild edible plants through a contest of desserts prepared from any of the 14 studied plants [Figure 1].

This study was part of the project “Caatinga Plant Resources: Use, Diversity and Conservation”, which was approved by the Ethics Committee for Research Involving Human Subjects of the Health Sciences Center of Pernambuco Federal University (register number 238–06).

## Results

### a) Knowledge and use of native wild edible plants

The most and the least known items collected were identified, as also were the most and the least used items in the past and in the present. Of the 14 edible items addressed in our survey, 7 were reported to be known by all of the interviewees, whereas the manofê (*Mandevilla tenuifolia*), whose tubercles are consumed, was the least known plant and was only reported by half of those individuals interviewed. A total of 3 of the edible items (coco catolé (*Syagrus cearensis*), cladode of facheiro (*Pilosocereus pachycladus*) and imbu fruit (*Spondias tuberosa*)) had been consumed at least once during the lifetimes of all interviewees. However, none of the edible items was currently consumed by all the respondents. The most-consumed item was the imbu fruit (*S. tuberosa*), associated with a current consumption rate of 97%.

Additionally, among the 14 edible items, there were five that had been only barely tried by more than 20% of those surveyed (*Crateva tapia*, *Cereus jamacaru*, *Neocalyptrocalyx longifolium*, *Eugenia pyriformis* and *Hymenaea courbaril*). Individuals who did not consume a particular edible item reported that they had abstained from doing so mainly due to unpleasant morphological or chemical characteristics of the plant, specifically taste and smell (61%).

Among the reasons cited for not having tried any of the 14 edible items in the survey, the most common, mentioned by 46% of the interviewees, was that their lack of interest might have been due to not knowing that such plants could be used for human consumption, followed by not considering that such plants should be used as food (39%).

When asked if these edible items were consumed with the same frequency in the past or if their consumption had varied over time, most of the interviewees (62%) answered that they currently consume less wild plants than in the past, except for the imbu fruit (*S. tuberosa*) or the facheiro cladode (*P. pachycladus*). The reasons mentioned for eating these edible items less now than in the past were having no need for these plants now (32%) and the difficulty of finding the plants in the wild (23%).

We evaluated whether there was a relationship between the age of people interviewed and the number of edible items they knew, and we found that these two variables were positively related (rs = 0.38; p = 0.0175). We also checked whether there was a relationship between knowledge and past and current use and found that these parameters were not significant (p = 0.1766), but there was a significant relationship between knowledge and past use (rs = 0.56; p = 0.0362). In addition, we determined whether there was a relationship between the age of the people interviewed and the number of edible items used in the past and currently and found that there was a relationship with past use, but not with current use (adjusted R^2^ = 21.40% for past use; p < 0.05). We found a significant, although weak, inverse relationship between current use and monthly family income (rs = −0.38; p < 0.05) (Table 3). Furthermore, we evaluated whether there were differences associated with the ages of those interviewed and individual income and found that these variables were correlated (rs = 0.68; p < 0.0001).

**Table 3:** Relationships of age, monthly family income and monthly individual income with past and current use of the native wild edible plants from a local community in NE Brazil

	**Relationship of past use vs.**		**Relationship of current use vs.**	
	**p value**	**Index**	**p value**	**Index**
Age	0.0018*	R^2^aj. = 21.40%	0.0720	R^2^aj. = −6%
Family income	0.7680	rs = −0.05	0.0172*	rs = −0.38
Individual income	0.5104	rs = 0.11	0.0750	rs = −0.29

*p < 0.05.

We also analyzed whether there was a relationship between consumption (past or current) and the gender of those interviewed as well as with their current and past occupations and found that none of these six pairs of variables were statistically related. However, in all cases, there was more consumption of the edible items in the past than there was currently. We also found that men had consumed and continued consuming more edible items than women.

It was also observed that individuals who had worked in agriculture in the past consumed and still consume slightly more edible items than those with non-agricultural occupations and that people currently working in agriculture had consumed fewer native wild plants in the past than people not working in agriculture. However, people working in agriculture now consume more of these edible items than those in non-agricultural occupations.

We found no significant differences between the number of edible items known by farmers and the number known by non-farmers (Kruskal-Wallis p-value = 0.7962). We also found no correlation between gender and occupation (G-test (Yates) = 1.0333, p = 0.3094).

During the informal conversations we engaged in as part of this study, we observed that the surveyed individuals tended to use plants that are associated with diverse forms of preparation, those with many uses and particularly those that present medicinal properties more often. Next, we found that the correlation between use and the number of food preparation methods associated with a plant was significant (rs = 0.75 for current use, rs = 0.81 for past use; p < 0.05) as it was the correlation between past use and the number of uses (rs = 0.60; p < 0.05). There was no significant relationship between current consumption and the number of uses or between consumption and the medicinal use of these species (Table 4).

**Table 4:** Relationships of the number of ways to prepare a plant, the number of uses and medicinal use with consumption (past and current) of the native wild edible plant species from a local community in NE Brazil

	**Preparation methods**		**Use**		**Medicinal use**	
	**p value**	**Index**	**p value**	**Index**	**p value**	**Index**
Past consumption	0.0004*	rs = 0.81	0.0222*	rs = 0.60	0.8517	rs = 0.05
Current consumption	0.0020*	rs = 0.75	0.0781	r (Pearson) = 48.58%	0.8706	rs = −0.05

* p < 0.05.

### b) Management of native wild edible plants

The most common preparation method was *in natura* (93%) consumption, except for the facheiro cladode (*P. pachycladus*), which is mainly consumed as sweet jam. When asked where the most common location to collect these plants was, most of the surveyed individuals agreed that most of the edible items (79%) were collected mainly in the mountainous region of the community.

The majority of interviewees affirmed that the lower region of the community was the preferred area for collection (42%) because the community inhabitants commute through this area, and the collection of edible items here does not require a detour but can be carried out while in transit (33%). However, this response was obtained for many but not for all of the species because some of them can only be found in the mountainous region, and if people want to consume them, it is necessary to go to that region to find them.

Those interviewed were also asked if they leave their houses with the specific goal of collecting wild edible plants. The majority responded “no” (82%) and reported that wild edible plants are consumed as they are found, which usually occurs when performing other activities, like preparing the land for sowing or grazing their animals. The interviewees were also asked if any of the 12 wild edible species were cultivated, and the response was mostly negative (87%), with the exception of *S. tuberosa* (imbu) and *Crateva tapia* L. (trapiá); some people indicated that these species are planted and watered until they reach a size at which they can survive on their own.

The respondents were asked what they would change if they could hypothetically change something about these edible items. The majority responded that they would not change anything (75%). The second most common response (14%) was that they would change some morphological or chemical characteristic (e.g. they would like them to be sweeter, larger or to have a more pleasant smell).

Another hypothetical question posed in the interviews was related to what the individuals would do if they were planting crops somewhere and found any of the wild edible plants included in the study. The respondents indicated that they would not remove them (69%), except for the incó (*N. longifolium*) and the mandacaru (*C. jamacaru*), which according to those interviewed, would be removed because they do not provide sufficient benefits but occupy space and could be associated with problems (specifically, in the case of mandacaru, regarding the presence of spines). We found a significant correlation between age and the number of plants that the interviewees would remove (not tolerated) if they were clearing an area for planting (rs = 0.36; p < 0.05). Additionally, we found an inverse correlation between family income and the number of plants that individuals would tolerate in the area (re = −0.35; p < 0.05), as shown in Table 5.

**Table 5:** Relationships of age, family income and individual income with parameters indicative of the degree of management of the native wild edible plants from a local community in NE Brazil

	**Relationship with ‘leaving to collect items’**		**Relationship when cleaning an area for planting vs.**			
			**Would leave it**		**Would remove it**	
	**p value**	**Index**	**p value**	**Index**	**p value**	**Index**
Age	0.1861	rs = −22.00	0.7565	R^2^aj. = −2.43%	0.0702	r(Pears) = 29.3%
Family income	0.0295*	rs = −0.35	0.8891	rs = 0.020	0.571	rs = 0.09
Individual income	0.0509	rs = −0.32	0.9638	rs = 0.007	0.236	rs = 0.19

*p < 0.05.

In addition, we analyzed the relationship of gender and current and past occupation with the proportions of people who purposely leave their homes to collect edible plants and those who do not, as well as with the proportions of people who would tolerate the plants in an area and those who would not tolerate them. These variables are only correlated with actual occupation for two management parameters (χ^2^ = 4.261 for leaving exclusively to collect, χ^2^ = 7.711 for the proportion of people who would tolerate these species and those who would not tolerate them in their lands; p < 0.05 both cases). This means that farmers leave exclusively to collect more plants than people who are not farmers and, hypothetically, that farmers are more tolerant of these plants than non-farmers. Despite not finding significant differences, it was observed that men tend to collect more plants than women and tend to tolerate these plants more than men.

## Discussion

### a) Knowledge and use of native wild edible plants

We found a strong correlation between the more known edible items and their presence in the lower mountainous zone. This coincides with the results obtained by Ladio et al. [30], who observed that the plants found at closer locations are the most commonly used.

This usage pattern seems to be related to the optimal foraging theory [31], according to which humans, as well as other animals, consider the costs and benefits associated with collecting resources in different environments and/or ecosystems, such that if a collecting location is too distant (increased cost) but offers many resources (increased benefit), then it could be worth travelling to the site and the consequent energy expenditure. However, if a collecting site is distant and does not offer a great variety or availability of resources, it would tend to be poorly visited for the extraction of those resources [32,33], which was found to be the case in our study area.

A positive significant correlation, albeit a weak one, was observed between knowledge about the edible items and a plant exhibiting many uses, which is a result also reported by other authors, such as Ogle et al*.*[34].

The current use of a plant is not necessarily related to knowledge about it [1,9,32,35] (e.g. the facheiro fruit (*P. pachycladus*), the imbu potato (*S. tuberosa*), the mandacaru (*C. jamacaru*) and the trapiá (*C. tapia*) are known by all those interviewed but are not currently consumed). These findings indicate that in addition to knowledge, there are other important factors involved in determining the consumption of a plant, such as ideas that people have about these plants [36]. An example is the case of the imbu (*S. tuberosa*). During the community events, some people mentioned not eating it in large amounts, not because they dislike its flavor, but because they consider its consumption to constitute a possible conservation risk for this plant. For other plants, such as the mandacaru (*C. jamacaru*), it can be determined that the lack of consumption is likely due to cultural reasons rather than biological ones. For example, people in Carão refer to toad eggs, which are covered by a foamy proteic layer to protect them from potential predators, as “toad foam” [37]. This plant might also not be used because some people in this community consider its consumption to be a health risk stemming from the false belief that ingesting its seeds causes appendicitis. Thus, factors such as these could lead to the creation of a food taboo, according to the hypothesis proposed by Meyer-Rochow [38]). These differences could be due to the strong dependency relationship among the members in the community studied by Ladio and Lozada [31], as well as to the nomadic lifestyle of their ancestors, which could have a strong influence on the way wild plants are used. This was the explanation offered by Reyes-García et al*.*[1] for a similar case in Bolivia.

With respect to the plants that were cited as only barely tried by the majority of those surveyed (i.e. trapiá (*C. trapia*), mandacaru (*C. jamacaru*) and incó (*N. longifolium*)), it was observed that sensorial and cultural factors, rather than biological ones, determine the use of these plants.

The most common reasons stated by the participants for not consuming some edible items were that they ignored the fact that the items were edibles or that they did not consider the items to be an edible item. In the same way, some authors [2,12] have cited a lack of knowledge as the main reason for not using an edible plant.

The majority of the interviewees indicated that they consume fewer of these plants at present than they did in the past, except for the imbu (*S. tuberosa*) and the facheiro cladode (*P. pachycladus*), for which there seems to have been no variation in consumption. According to the people in Carão, the use of these plants has declined because their consumption is not currently necessary, given the availability of more appealing edible plants that are easy to obtain.

According to Hardesty [39] and Castro and Begossi [40], under conditions of high resource abundance, human populations tend to prefer valued items, thus reducing the diversity of their diet, while under conditions of resource scarcity, there is a tendency to use a larger diversity of species to satisfy nutritional needs. Based on these findings, Ladio et al*.*[22] proposed a hypothesis, according to which the Mapuche community that was closer to urban centers and, thus, had more access to items would be expected to use a lower diversity of wild plants in comparison with the community located more distantly from urban centers.

The hypothesis proposed by Ladio et al. [30], regarding the relation between abundance and preference for consumption, despite not being supported in their study, could be related with the results found here. Hence, under scarcity conditions, as existed in the Carão community approximately 20 years ago, a greater diversity of edible species would have been used than at present because financial assistance programs have improved the quality of life of the inhabitants of this rural zone, as also found by Sieber et al. [26].

Similarly, Reyes-García et al*.*[1] and Ekué et al. [18] state that the ease of access of local communities to industrialized products induces losses of knowledge and traditional practices related to natural resources. Ngugi [8] affirms that communities with large agricultural lands tend to decrease their usage of wild plants.

The knowledge about edible items was directly correlated with age, indicating that older people tend to know native wild plants better, as has been reported in other studies [6,9].

However, even though many edible items are well known by elders, it is common that these are not used due to diverse health problems that prevent old people from obtaining or consuming them (e.g. due to the high sugar and fat content that people associate with these plants or the difficulty of eating them because of a lack of teeth).

Our results indicate that past consumption is related to age, which could be due to two factors: in the past, it was more common to eat these plants because of need and tradition compared to current times, as indicated by several respondents, and because older people have had more time to come to know and experiment with these plants compared to younger people.

Both old and young people currently consume approximately the same small amount of wild edible items. Other authors have reported that knowledge regarding these plants is related to age [6,9]; in our survey we found no association between consumption and age.

Both women and men use the native wild edible plants in similar ways, which may be due to similar activities being associated with both genders as has been reported in other studies [3,41]. Therefore, both women and men are in contact with the wild edible plants that are consumed, as will be discussed later, generally opportunistically. The number of uses was found to be related to past consumption, which was similarly reported by Nates et al*.*[42]. Likewise, Ladio et al. [30] reported a potentiating effect of a species’ versatility on its utilization, thus leading to the preservation of associated knowledge.

Here, we found results similar to those reported by Rivera et al. [43]: therapeutic use and consumption of edible items are not related.

### b) Management of native wild edible species

The fact that nine of the 14 edible items (64%) are consumed exclusively *in natura* could be related to the scarce interest that Carão inhabitants have regarding the usage of this kind of plant. If they were really interested in their utilization, it would be expected that they would explore diverse ways of using these edible plants, consuming them routinely. According to comments made by some members of the community, many of these plants were associated with more forms of preparation in the past, but because the tradition of using them has been lost, the associated knowledge regarding the ways to prepare them has been lost as well.

Based on the obtained responses, the region where these plants are most commonly collected is the mountainous region because some of the plants are only present there. However, the people interviewed affirmed that their preferred place to collect the plants is in the lower region because this is where they perform most of their activities, as has been reported in other studies [6,30].

The interviewees indicated that they did not specifically leave to collect most of the plants addressed in our survey, except in the case of the imbu fruit (*S. tuberosa*) and the facheiro cladode (*P. pachycladus*) to prepare sweet jams. Taken together with comments from some people with whom informal conversations took place, this indicates opportunistic consumption of these plants. This means that, except for the two above-mentioned items, most of these plants are collected and consumed when some other activity is taking place, for example, when people travel to corn, bean and manioc fields or when they bring animals to feed, as has been reported in other studies [3,44].

When the surveyed individuals were asked about the care received by these plants, they answered that most of them receive no care, which is a result consistent with the report of Agea et al*.*[19]. The exceptions are the imbu (*S. tuberosa*) and the trapiá (*C. tapia*) because the first plant is valued as a human and animal food source, and the second species, though not widely used as food, is valued because of the shade it provides. However, when the interviewees were asked about what they would do if they were planning to cultivate an area containing the investigated species, the majority indicated that they would not remove them because they provide food for people and other animals. A similar result was reported by Lins Neto et al*. *[11] with respect to imbu (*S. tuberosa*), the wood of which is not used because the species is more valued because of the services it provides when alive (human food, animal feed and shade) than those obtained when it is cut.

The four *in situ* management types previously described are practiced in the community. Tolerance is the most common and is employed for most of these plants (all except mandacaru (*C. jamacaru*) and incó (*N. longifolium*)); followed by selective collecting, which is presumably practiced on the facheiro cladode (*P. pachycladus*) and imbu tubercle and fruit (*S. tuberosa*); and, finally, protection and promotion are practiced mainly on imbu (*S. tuberosa*) and trapiá (*C. trapia*), according to Lins Neto et al*.*[11].

The fact that most of these practices are being used for very few of the species included in this study reflects the low level of appropriation of nature exhibited by the inhabitants of the rural community of Carão. In general terms, the type of management applied to the native wild edible plants is mostly incipient in the region, which has also been reported by Nascimento [5].

Family and individual income were weakly inversely related to collecting activity, as has also been observed in some studies [19]. Our results suggest that both young and old people collect these plants with similar, very low intensity, with the exceptions of facheiro cladode (*P. pachycladus*) and imbu fruit (*S. tuberosa*).

None of the management indicator variables were found to be related to gender or past occupation, but they were related to current occupation because farmers tend to leave specifically to collect slightly more plants than non-farmers, and farmers tend to tolerate edible plants in their plots more than non-farmers.

Results about current occupation can be explained by the fact that the great majority of non-farmers are older and receive pensions, thus, income can be confounding the association. Health condition of elders also can be a confounder as it prevents them from collecting these plants. Results about gender differ from findings of other authors [18,19].

The divergences between the results of the present study and those of Ekué et al. [18] and Agea et al*.*[19] could be due to the fact that men and women in the Carão community tend to perform the same activities, in the communities in Africa in which these teams of researchers worked, it is possible that activities are more differentiated by gender. However, because neither Ekué et al*.*[18] nor Agea et al*.*[19] made any mention of the tasks performed by men and women in the studied communities, there is no way to know whether these divergences are or are not caused by this factor.

## Conclusions

With respect to our stated hypothesis, knowledge regarding wild edible plants was related to age. However, neither use nor occupation showed relationships with knowledge of these plants. It was not possible to find relationships between age and actual usage. However, with respect to past usage, there is evidence indicating generalized abandonment of these resources. There was also no evidence of a relationship between use and gender or occupation. The most commonly observed management practice for these plants was tolerance, followed by selective collection.

We found that the conservation of these species is not endangered by its use, but perhaps by the deforestation of the ecosystems in which these plants grow, it would be possible and advisable to promote the consumption of such plants by the members of this community. We consider that the promotion of responsible use of them may lead to the conservation of knowledge while contributing to conservation, as been show by the works done by Altieri and others [45], who say the gathering of biologic resources for daily use is associated with the maintenance of a strong cultural tradition. In order to promote the consumption of these plants, it is important to start by teaching people the plant species that can be used for their alimentation, disprove the myths that exist about their use, and encourage diversification of uses, through the motivation for the invention of new ways of preparation. An example of how this can be done is through events such as the “Preserves Festival”.

Further, it is recommended to continue asking around the other factors that may determine the use of wild edible plants, since, as we have seen, at least in this community do not seem to be biological, but also cultural factors which determine the use of this type of plants.

## Competing interests

The authors declare that they have no competing interests.

## Authors’ contributions

All authors participated in the design of the study and writing the paper. All authors read and approved the final manuscript.
